# Sonographic characteristics of diffuse sclerosing variant of papillary thyroid carcinoma with histopathological correlation: a preliminary study

**DOI:** 10.1186/s13023-023-02867-3

**Published:** 2024-03-26

**Authors:** Wanying Li, Ying Wang, Luying Gao, Ruie Feng, Ke Lv, Xining Wu, Xiao Yang, Sheng Cai, Hongyan Wang, Jianchu Li

**Affiliations:** 1 Department of Ultrasound, State Key Laboratory of Complex Severe and Rare Diseases, Peking Union Medical College Hospital, Chinese Academy of Medical Sciences and Peking Union Medical College, Beijing, 100730 China; 2 Department of Pathology, Peking Union Medical College Hospital, Chinese Academy of Medical Sciences and Peking Union Medical College, Beijing, 100730 China; 3 Department of Health Management, Peking Union Medical College Hospital, Chinese Academy of Medical Sciences and Peking Union Medical College, Beijing, 100730 China

**Keywords:** Diffuse sclerosing variant of papillary thyroid carcinoma, Ultrasound, Histopathology, Thyroid nodules

## Abstract

**Background:**

Diffuse sclerosing variant of papillary thyroid carcinoma (DSVPTC) is a rare but high invasive subtype of papillary thyroid carcinoma, which mandates an aggressive clinical strategy. Few studies have focused on the sonographic characteristics of DSVPTC and the role of ultrasound in diagnosis and treatment of this variant remains unknown. This study aimed to identify and understand DSVPTC more accurately under ultrasound in correlation with pathology.

**Methods:**

The ultrasound characteristics and histopathologic sections of 10 lesions in 10 DSVPTC patients who underwent thyroid surgery at our center between 2014 and 2020 were reviewed and compared with 184 lesions in 168 classic variant of papillary thyroid carcinoma (cPTC) patients.

**Results:**

6 DSVPTC cases (60%) showed the “snowstorm” pattern on sonogram and 4 cases (40%) presented hypoechoic solid nodules only. Vague borders (100.0% vs. 18.5%, *P* = 0.019) and abundant microcalcifications (66.7% vs. 10.9%, *P* = 0.037) were more common in DSVPTC nodules than in cPTC nodules, corresponding to the infiltrating boundaries and numerous psammoma bodies under the microscope respectively. Most of the DSVPTC cases had a heterogeneous background (80%) and suspicious metastatic cervical lymph nodes (80%) on sonograms. All DSVPTC cases had histopathological metastatic cervical lymph nodes.

**Conclusion:**

The sonographic “snowstorm” pattern indicated DSVPTC with whole-lobe occupation. Hypoechoic solid nodules with vague borders and abundant microcalcifications on sonogram suggested DSVPTC lesion with an ongoing invasion. Regardless of which of the two sonograms was shown, the corresponding DSVPTC lesions were aggressive and required the same attention from the surgeons.

**Supplementary Information:**

The online version contains supplementary material available at 10.1186/s13023-023-02867-3.

## Background

Thyroid cancer is the most prevalent malignancy of the endocrine system, and approximately 90% are differentiated thyroid cancers [1]. Papillary thyroid carcinoma (PTC) is the main contributor [2]. There are many histological subtypes of PTC, such as the classic, follicular, solid, tall cell and diffuse sclerosing variant. Classic variant of papillary thyroid carcinoma (cPTC), although the largest, is relatively indolent. However, some subtypes are biologically aggressive. Diffuse sclerosing variant of papillary thyroid carcinoma (DSVPTC) is relatively rare but invasive, accounting for 0.7-6.6% of PTCs [3].

DSVPTC was first described by Vichery et al. [4] in 1985, and was formally listed in the variants of PTC by the WHO in 1988 [5]. It has a preponderance for younger entity, especially for females [3]. A study suggested that DSVPTC was a major subtype of PTC in people under 20 years old [6]. Extrathyroidal extension and distant metastasis were more commonly seen in DSVPTC than in well-differentiated PTC [7]. The American Thyroid Association Risk of Recurrence stratification classified DSVPTC as intermediate risk [8], indicating a poor prognosis, which mandates an aggressive clinical strategy [9]. Additionally, there were cases reported that the preoperative diagnosis of DSVPTC was challenging [10], and some cases could be confused with chronic thyroiditis and delay the diagnosis and treatment [11]. Therefore, analyzing the sonograms of DSVPTC is necessary.

Ultrasound is the most common modality for evaluating thyroid lesions. The biological nature of the lesions is assessed mainly based on the composition, echogenicity, shape, margin and echogenic foci on sonogram, which is of instructional clinical value. For nodules, systematic ultrasound evaluation systems, such as the American College of Radiology Thyroid Imaging, Reporting and Data System (ACR TI-RADS), have been developed and widely used. However, only a few studies have examined the imaging manifestations of DSVPTC. The largest study included 8 cases [12]. Although it was previously shown that “snowstorm” pattern was a special sonographic sign of it [12, 13], the sonograms of DSVPTC had not been compared with histopathology and the clinical significance of these characteristics remains unknown. Our study retrospectively analyzed the sonograms of DSVPTC with pathology correspondence over the past 7 years. The aim is to summarize the sonographic characteristics of DSVPTC, so as to help preoperative diagnosis and guide clinical practice.

## Methods

### Patients

Our study was approved by the Institutional Review Board of our center. We retrospectively reviewed all patients with PTC confirmed by surgery in our hospital between January 2014 and December 2020. Among this initial cohort, only patients who met the following criteria were included: [1] total or partial thyroidectomy was performed; and [2] histological diagnosis of DSVPTC was confirmed. Two patients were excluded for incomplete preoperative sonographic data. Finally, 10 DSVPTC patients were included in our study. Measurement of serum thyroxine (T4), free T4 (FT4), triiodothyronine (T3), free T3 (FT3), thyroid stimulating hormone (TSH), thyroid peroxidase antibody (TPOAb) and thyroglobulin antibody (TgAb) were performed for all 10 patients before the operation.

We sequenced all the cPTC patients confirmed by pathology according to operation time in our hospital during the same period, and selected 200 as controls using systematic sampling. After eliminating cases by the same evaluation criteria, 168 cPTCs with 184 nodules were enrolled.

### Thyroid ultrasound examination and retrospective evaluation

Experienced radiologists performed ultrasound examination using IU 22 or EPIQ 7 (Philips Medical Systems, Bothell, WA) equipped with 5–12 MHz linear transducers. Two radiologists with 3 and 6 years of experience in thyroid imaging reviewed the scanning videos independently, and images served as auxiliary when the video provided limited information or was vacant. Ambiguities were resolved by a radiologist with 30 years of experience in thyroid imaging.

The sonographic characteristics of DSVPTC were analyzed based on two aspects including parenchyma and nodule. For the first aspect, the gland size, echogenicity, blood supply, and the presence of the “snowstorm” pattern, nodules and suspicious metastatic cervical lymph nodes were reviewed. The gland size categories were normal and enlarged. The anteroposterior diameter of any lobe greater than 2 cm was regarded as enlarged [14]. The echogenicity of the gland was classified as homogeneous and heterogeneous with reference to the normal submandibular gland [15]. The blood supply of the gland was labeled as normal and “rich”. The term “rich” indicated increased color signals at the level of peripheral thyroid arteries and presence with patchy distribution in the lobe [16]. The “snowstorm” pattern was defined as microcalcifications (diameter less than or equal to 1 mm and without shadowing) distributed diffusedly in at least one entire lobe [17]. Cervical lymph nodes with any of the following features were considered suspicious metastatic [18]: [1] loss of central hilar echo, [2] cystic change, [3] calcification, [4] cortical hyperechogenicity, and [5] increasing and irregularly distributed vascularity.

For the cases with nodules, we also analyzed the size, location, shape, margin, composition, echogenicity, echogenic foci, TI-RADS level, border and the presence of abundant microcalcifications. The largest diameter of the nodule was used to assess the size. The borders with well-defined parts constituting more than 75%, 51-75%, 25-50%, or less than 25% were assigned scores from 1 to 4, respectively. When the sonogram illustrated more than ten microcalcifications in the nodule, “abundant” was used for description. Adler’s semiquantitative method was used to classify vascularity [19]. Other conventional evaluation indices were evaluated according to the ACR TI-RADS (the 2017 edition) [20].

### Pathology

Pathology slides were made by formalin-fixed paraffin-embedded tissue blocks. One experienced pathologist reviewed the hematoxylin-eosin (H&E) stained sections of all cases together with one imaging reviewing radiologist to refer to ultrasound information. The classification of thyroid cancer was based on the pathological diagnosis of malignant tumors established by the WHO. The size (maximum diameter), location of the lesions, the presence of Hashimoto’s thyroiditis and metastatic cervical lymph nodes were recorded. Lymph node metastasis was analyzed as a binary and a continuous variable.

### Statistical analysis

Data were analyzed using SPSS software version 21.0 (SPSS Inc., Chicago, IL). The normality of distribution was evaluated by the Shapiro‒Wilk test or Kolmogorov‒Smirnov test. Standard’s *t* test or the Mann‒Whitney test was used for continuous variables, and the chi-square test or Fisher’s exact test was used for categorical variables. All statistical tests were bilateral, and a *P*-value less than 0.05 was considered statistically significant.

## Results

### Clinical findings

The patients included 9 (90%) females and 1 (10%) male with a median age of 36 years old (range: 16–67). 3 patients had a palpable local cervical mass, 2 patients complained of neck enlargement, and 5 visited the hospital with no discomfort but had an abnormity in their regular checkup (1 for thyroid function test and 4 for neck ultrasound). None of our patients had a family history of thyroid cancer or radiation exposure. 1 patient had lung cancer and received pulmonary wedge lobectomy a year ago. In the preoperative thyroid function test, TSH was high in 3 patients (normal range: 0.38–4.34 µIU/mL). TPOAb was positive in 3 patients (normal range: <34 IU/mL), and TgAb was positive in 4 patients (normal range: <115 IU/mL). Only 4 patients had all normal indices.

### Ultrasound findings

The sonographic findings of DSVPTC and cPTC are summarized in Table 1. Among these, the heterogeneous background (80%, *P* = 0.004) and suspicious metastatic cervical lymph nodes (80%, *P* = 0.003) were common in DSVPTC. In addition, the “snowstorm” pattern (60%, *P* < 0.001) and rich underlying flow signals (60%, *P* < 0.001) were more frequently seen in DSVPTC as well. More cases without nodules were seen in DSVPTC (*P* < 0.001).

**Table 1:** Sonographic characteristics between patients with DSVPTC and cPTC

	DSVPTC (%) (*n* = 10)	cPTC (%) (*n* = 168)	*P*-value
Thyroid enlargement	2 (20.0)	2 (1.2)	0.016
Underlying echogenicity			0.004
Homogeneous	2 (20.0)	113 (67.3)	
Heterogeneous	8 (80.0)	55 (32.7)	
Underlying flow signals			< 0.001
Normal	4 (40.0)	155 (92.3)	
Rich	6 (60.0)	13 (7.7)	
“Snowstorm” pattern	6 (60.0)	4 (2.4)	< 0.001
Nodules			< 0.001
With nodules	6 (60.0)	166 (98.8)	
Without nodules	4 (40.0)	2 (1.2)	
Suspicious metastatic lymph node	8 (80.0)	51 (30.4)	0.003

According to the main grayscale echogenicity features, DSVPTCs were divided into diffuse and focal groups. The diffuse group included 6 cases with a “snowstorm” pattern on sonograms. 4 cases were listed in the focal group for showing nodules only on sonograms, without a “snowstorm” pattern. Disparities in other characteristics were not found in the diffuse and focal groups (Table 2).

**Table 2:** Clinical, sonographic and pathological characteristics between DSVPTC cases in diffuse and focal group

	DSVPTCs in diffuse group (*n* = 6)	DSVPTCs in focal group (*n* = 4)	*P*-value
Age (years)	29.8 ± 9.7	45.3 ± 18.3	0.117
Sex			0.400
Male	0	1	
Female	6	3	
Ultrasound findings			
Thyroid enlargement	1	1	1.000
Heterogeneous background	6	2	0.133
Abundant flow signals	5	1	0.190
Suspicious metastatic cervical lymph node	5	3	1.000
Multifocality	4	3	1.000
Hashimoto’s thyroiditis	5	2	0.673
Metastatic cervical lymph nodes			
Cases	6	4	1.000
Number	23.3 ± 16.0	14.3 ± 10.1	0.347

**Fig. 1:**
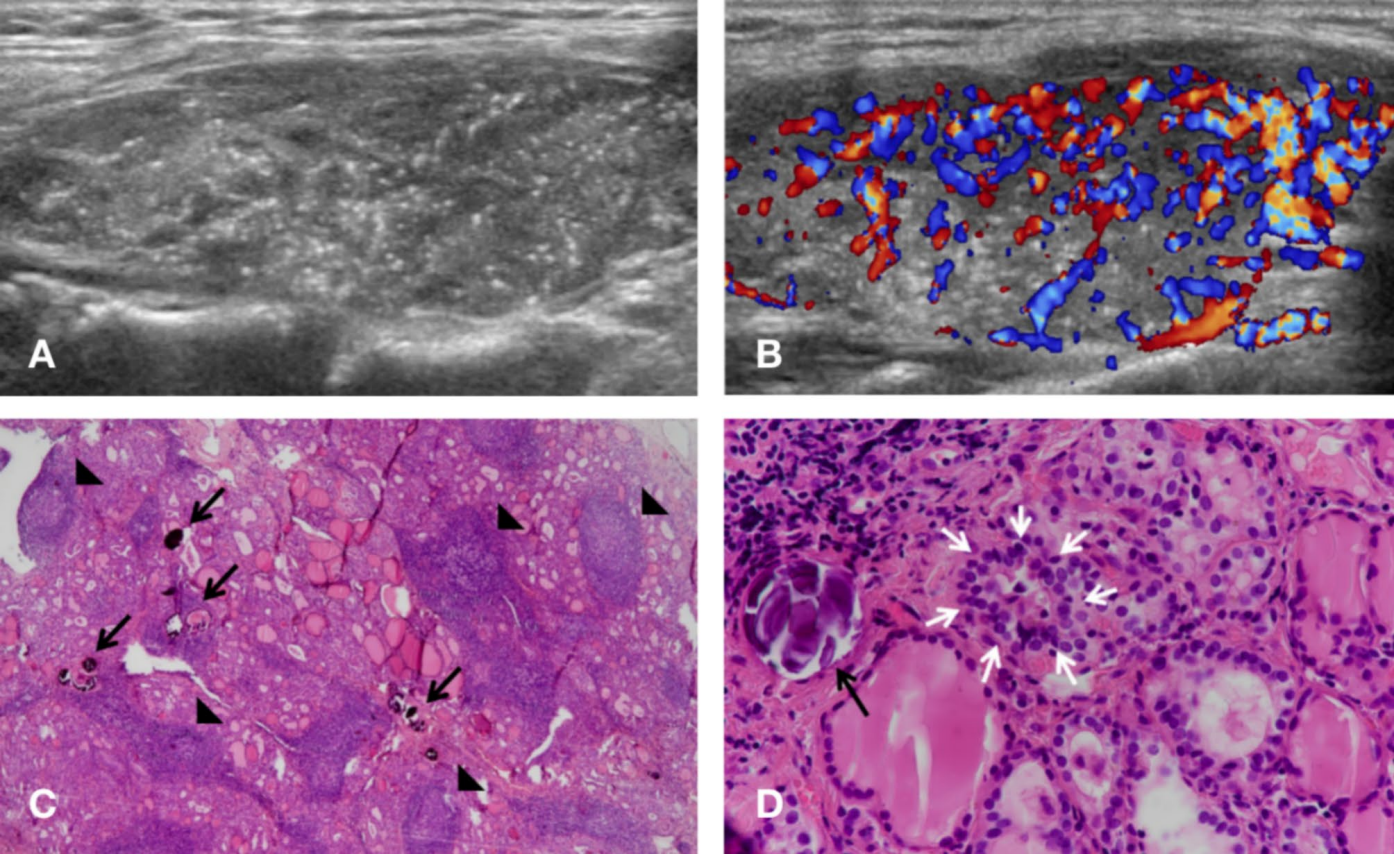
Ultrasonographic and pathologic findings of DSVPTC in a 35-year-old woman (Case 4). **(A)** Numerous microcalcifications in the whole right lobe like “snowstorm” without nodules in the longitudinal section of the thyroid. **(B)** Rich blood flow signals in the right lobe in color doppler flow imaging. **(C)** Lymphocytic follicles (black arrowheads) with numerous tumor cells and psammoma bodies (black arrows) in histopathology section (H&E stain, ×40). **(D)** One tumor nest (white arrows) and a concentric calcified psammoma body (black arrow) (H&E stain, ×400)

**Fig. 2:**
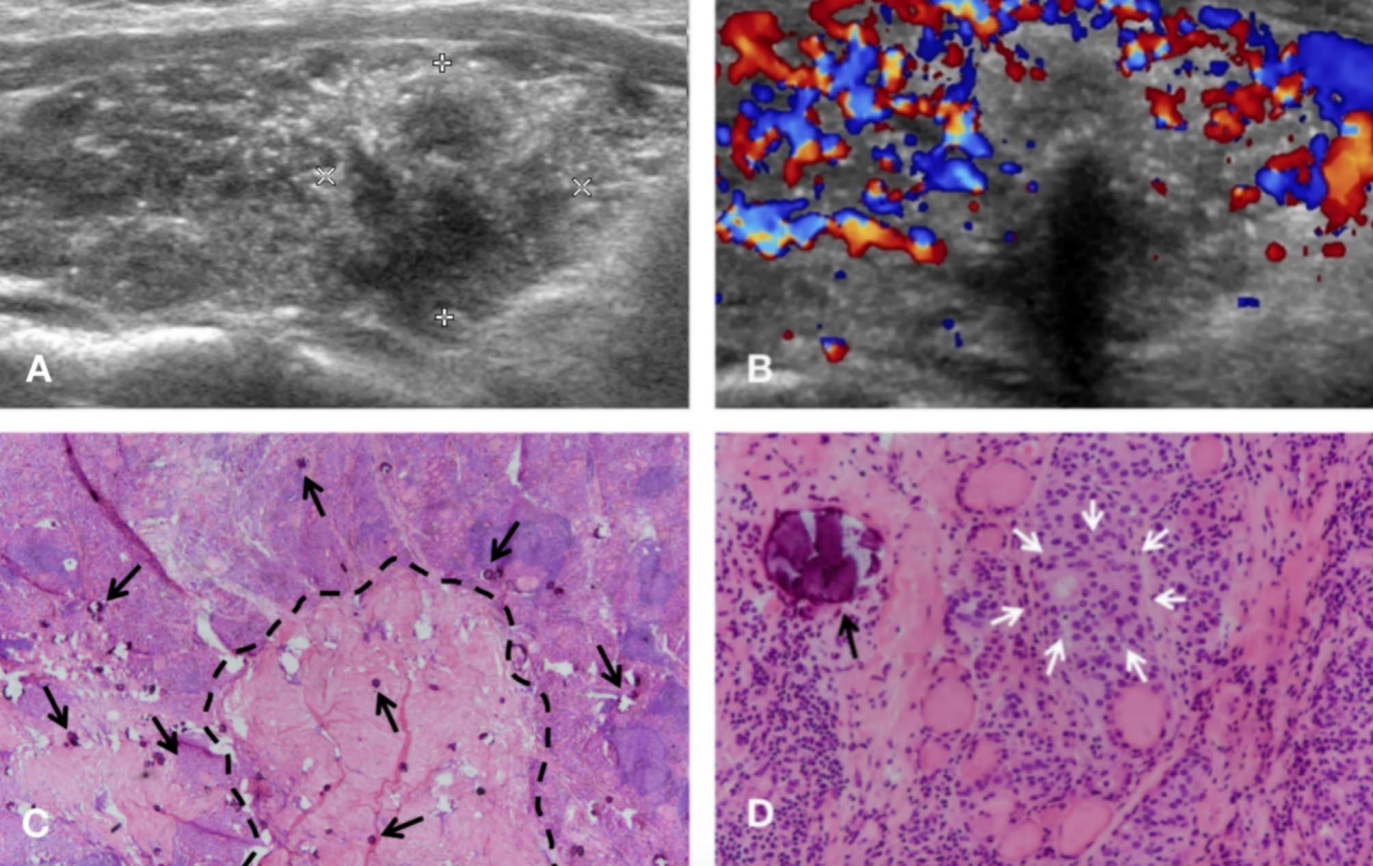
Ultrasonographic and pathologic findings of DSVPTC in a 36-year-old woman (Case 6). **(A)** The “snowstorm” pattern in the right lobe with a hypoechoic nodule of vague border in the longitudinal section of the thyroid. **(B)** Abundant flow signals in parenchyma of the right lobe and scarce signals inside the nodule in color doppler flow imaging. **(C)** A fibrotic nodule (broken black line) corresponding to the sonographic hypoechoic nodule with many psammoma bodies inside and outside the nodular area (black arrows) (H&E stain, ×20). **(D)** One tumor nest (white arrows) and a concentric calcified psammoma body (black arrow) (H&E stain, ×400)

In the diffuse group, one case presented the “snowstorm” pattern in the bilateral lobes, while others presented in unilateral lobe (Fig. 1). The heterogeneous background was widely seen in all 6 cases (100%), and 5 cases (83.3%) had rich flow signals. 2 cases (33.3%) were accompanied by hypoechoic solid nodules in the lobe with the “snowstorm” pattern (Fig. 2), one of which was multiple.

**Fig. 3:**
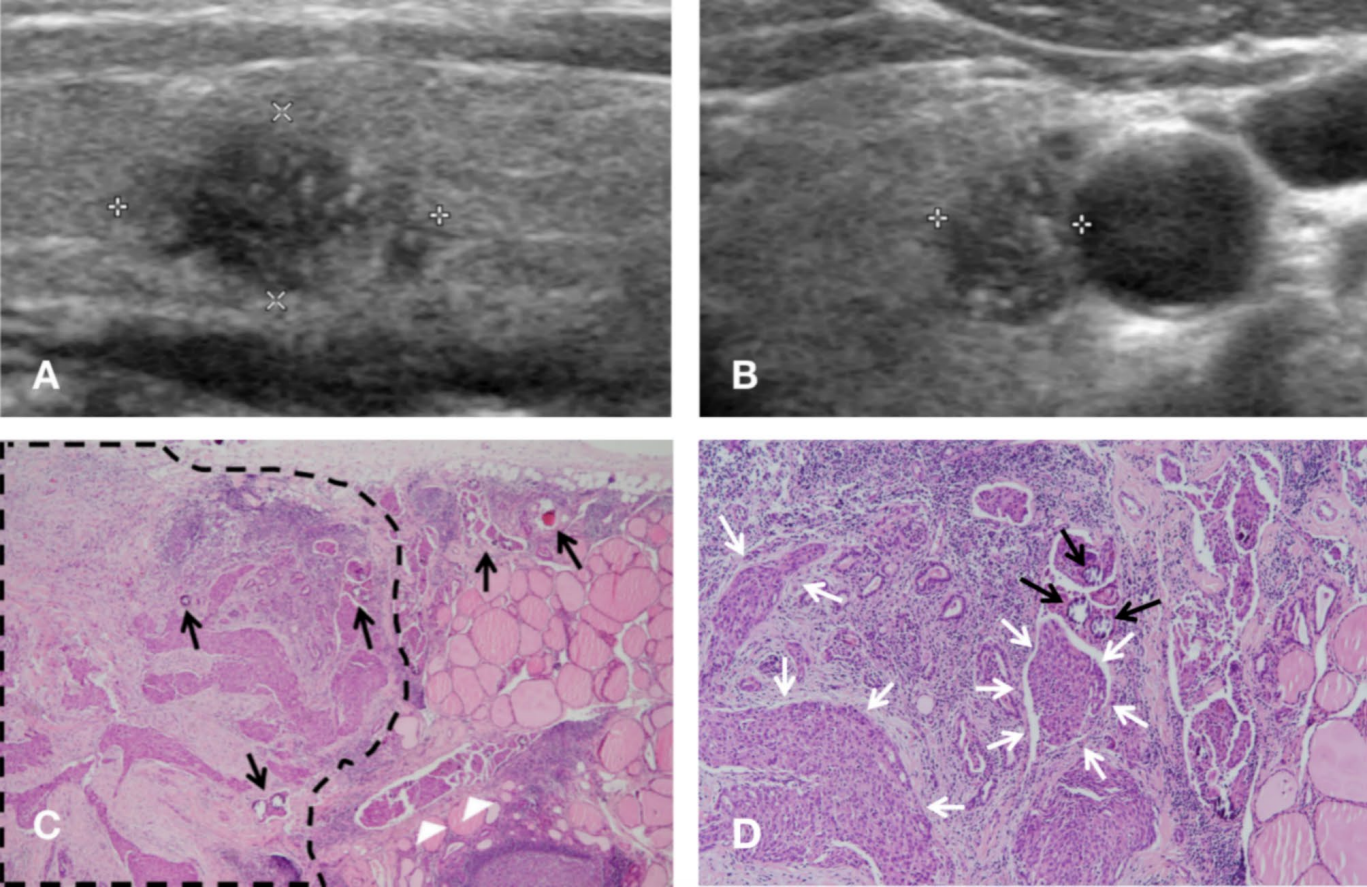
Ultrasonographic and pathologic findings of DSVPTC in a 26-year-old woman (Case 9). **(A)** A hypoechoic nodule with vague border and multiple microcalcifications in the left lobe with homogenous background in longitudinal section of the thyroid. **(B)** Transverse section of the same nodule. **(C)** Tumor cells clustering in one place (broken black line) and invading surrounding parenchyma with several psammoma bodies (black arrows) and a lymph vessel with tumor emboli (white arrowheads) in histopathology section (H&E stain, ×20). **(D)** Scattered psammoma bodies (black arrows) and tumor nests (white arrows) (H&E stain, ×100)

In the focal group, only 1 case had more than one hypoechoic nodule and we took the largest nodule for analysis. All 4 nodules shared the malignant appearance for solid composition, irregular margin and microcalcifications on sonogram (Fig. 3); however, only three of these were verified as DSVPTC lesions by pathology (DSVPTC nodules), and the other was a fibrotic nodule (see ultrasonographic images of other cases in Additional file 1).

Compared with cPTC nodules, 3 DSVPTC nodules in the focal group differed only in border (100.0% vs. 18.5%, *P* = 0.019) and abundance of microcalcifications (66.7% vs. 10.9%, *P* = 0.037). Taking a score of 4 as the vague border, we found that DSVPTC nodules were more likely to be characterized by a vague border combined with abundant microcalcifications than cPTC nodules (66.7% vs. 1.1%, *P* = 0.001). Significant differences were not found in other sonographic features, even in TI-RADS level (*P* = 1.000) (Table 3).

**Table 3:** Sonographic characteristics between DSVPTC nodules and cPTC nodules in focal group

	DSVPTC’s (*n* = 3)	cPTC’s (*n* = 184)	*P*-value
Size	1.1 ± 0.3	1.1 ± 0.7	0.493
Location			1.000
Left lobe	1	92	
Right lobe	2	92	
Composition			1.000
Cystic or almost completely cystic	0	0	
Spongiform	0	0	
Mixed cystic and solid	0	2	
Solid or almost completely solid	3	182	
Echogenicity			0.167
Anechoic	0	0	
Hyperechoic or isoechoic	0	5	
Hypoechoic	2	174	
Very hypoechoic	1	5	
Shape			0.858
Wider-than-tall	2	82	
Taller-than-wide	1	102	
Margin			1.000
Smooth or Ill-defined	0	29	
Lobulated or irregular	3	149	
Extra-thyroidal extension	0	6	
Echogenic foci			0.630
None or large comet-tail artifacts	0	48	
Macrocalcifications	0	7	
Peripheral (rim) calcifications	0	3	
Punctate echogenic foci	3	126	
TI-RADS			1.000
4	0	20	
5	3	164	
Border			0.019
1	0	37	
2	0	47	
3	0	66	
4	3	34	
Abundant microcalcifications	2	20	0.037
Vessel number			0.380
Grade 0	0	27	
Grade 1	0	30	
Grade 2	3	78	
Grade 3	0	49	

80% DSVPTC cases had suspicious metastatic cervical lymph nodes, significantly more than that of cPTCs (30.4%). Among these lymph nodes, loss of central hilar echo was common (87.5% vs. 68.6%, *P* = 0.499), and increasing and irregularly distributed vascularity was not observed. In addition, cystic change (12.5% vs. 21.6%, *P* = 0.904), cortical hyperechogenicity (12.5% vs. 11.8%, *P* = 1.000), calcification (37.5% vs. 39.2%, *P* = 1.000) and abundant microcalcification (12.5% vs. 5.9%, *P* = 0.451) could also be the sonographic manifestations of metastatic cervical lymph nodes of DSVPTC, which showed no disparity when compared with cPTCs.

### Pathological findings in association with ultrasound

Pathology demonstrated that DSVPTC lesions invaded the entire lobe (4 involving the bilateral lobes and 6 involving the unilateral lobe) in all 10 cases. Dense sclerotic fibrosis and obvious lymphocyte infiltration induced a heterogeneous background on sonogram. In the diffuse group, tumor cells and psammoma bodies were diffusely distributed in the whole lobe in 5 cases and unevenly spread with multiple gathering areas in 1 case. The “snowstorm” pattern was correlated with numerous psammoma bodies. With respect to 2 hypoechoic nodules in the diffuse group, one referred to the tumor cells cluster, and the other was a fibrotic nodule. In the focal group, 3 nodules were verified as gathering areas of tumor cells and psammoma bodies without capsule, corresponding to the infiltrative boundary on sonogram. The last nodule was a fibrotic nodule accompanied by scattered psammoma bodies in the DSVPTC lesion of the whole-lobe scale. The specific pathologic information in association with ultrasound of these 10 cases is summarized in Table 4. The incidence rates of DSVPTC with Hashimoto’s thyroiditis and cervical lymph node metastasis were 70% and 100% respectively, which were higher than those of cPTC (31.0%, 56.5%, *P* = 0.028, 0.006).

**Table 4:** Clinical, laboratory, imaging and related pathologic findings in DSVPTC

Case	Age	Sex	Preoperative thyroid function	Operation way	Background		Nodule		Metastatic lymph node	
					“Snowstorm” pattern on sonogram	Pathology	Ultrasound	Pathology	Ultrasound	Pathology
1	16	F	Normal	Total thyroidectomy + LND	Bilateral lobes	Diffused distribution of tumor cells and psammoma bodies in bilateral lobes	-	-	+	+
2	38	F	TSH above the normal range	Total thyroidectomy + LND	Left lobe	Diffused distribution of tumor cells and psammoma bodies in the left lobe and scattered distribution in the right lobe	-	-	+	+
3	19	F	Normal	Total thyroidectomy + LND	Left lobe	Diffused distribution of tumor cells and psammoma bodies in the left lobe	One nodule in the right lobe	One localized cPTC lesion in the right lobe	+	+
4	35	F	TgAb positive	Right thyroidectomy + LND	Right lobe (a few microcalcifications in the left lobe)	Diffused distribution of tumor cells and psammoma bodies in the right lobe	-	-	+	+
5	35	F	TPOAb positive	Total thyroidectomy + LND	Right lobe	Scattered distribution of tumor cells and psammoma bodies with lymph vessel tumor emboli in the right lobe	Multiple nodules in the right lobe	Gathering areas of tumor cells and psammoma bodies	+	+
6	36	F	TSH above the normal range, TgAb and TPOAb positive	Total thyroidectomy + LND	Right lobe	Diffused distribution of tumor cells and psammoma bodies in the right lobe	One nodule in the right lobe	A fibrotic nodule	+	+
7	35	F	TSH above the normal range, TgAb and TPOAb positive	Total thyroidectomy + LND	No	Scattered distribution of tumor cells and psammoma bodies in bilateral lobes, decreasing with distance from the nodule in the right lobe with lymph vessel tumor emboli	One nodule in the right lobe	Gathering area of tumor cells and psammoma bodies	+	+
8	67	F	TgAb positive	Total thyroidectomy + LND	No	Scattered distribution of tumor cells and psammoma bodies in the right lobe and sporadic tumor cells in the left lobe	Multiple nodules in the right lobe	Gathering areas of tumor cells and psammoma bodies in the right lobe	+	+
9	26	F	Normal	Left thyroidectomy + LND	No	Scattered distribution of tumor cells and psammoma bodies in the left lobe, decreasing with distance from the nodule with lymph vessel tumor emboli	One nodule in the left lobe	Gathering area of tumor cells and psammoma bodies in the left lobe	-	+
10	53	M	Normal	Total thyroidectomy + LND	No	Scattered distribution of tumor cells and psammoma bodies in the left lobe	One nodule in the left lobe	A fibrotic nodule in the left lobe	+	+
F: female; M: male; LND: lateral neck dissection										

## Discussion

We retrospectively reviewed the preoperative sonograms of 10 lesions in 10 DSVPTC patients related to histopathology compared with 184 leisons in 168 cPTC patients in the same period. Our results demonstrated that although all 10 DSVPTC lesions involved the whole lobe in pathology, only 6 (60%) appeared on sonogram in the form of “snowstorm” pattern. The other 4 (40%) presented as hypoechoic solid nodules with vague borders and abundant microcalcifications (3 were DSVPTC nodules, 1 was a fibrotic nodule). Pathologically, abundant microcalcifications on sonogram were associated with numerous psammoma bodies, and the vague borders of DSVPTC nodules were caused by infiltration into surrounding parenchyma. Additionally, the heterogeneous background (80%) and suspicious metastatic cervical lymph nodes (80%) were also common.

Based on our results, not all DSVPTC lesions with whole lobe encroachment exhibited the “snowstorm” pattern on sonogram. Only 6 DSVPTC lesions pathologically showing diffuse or multifocal tumor cells and psammoma bodies throughout the lobe had that pattern. Lesions in the focal group showed scattered distribution of relatively few psammoma bodies and tumor cells in the parenchyma. Considering that all DSVPTC lesions had pathologically total lobular involvement, we infer that the “snowstorm” pattern can be observed only when psammoma bodies of the lesion are densely distributed, which is consistent with the view of Wang Y et al. that >/= 5 psammoma bodies per ×200 field of microscope might be visible by ultrasound [21]. Previous studies have found that the occurrence rate of the “snowstorm” pattern in DSVPTC was approximately 83%∼100% [12, 13, 22, 23]. In our study, the rate was 60%. We believe the discrepancy is due to the development of ultrasound technology and the improvement of public health awareness, which facilitate the detection of nodular DSVPTC, leading to a decreasing proportion of “snowstorm” patterns.

We found that DSVPTC lesions presenting as hypoechoic solid nodules were aggressive with an ongoing invasion. Among the hypoechoic solid nodules in the focal group, the locations of 3 nodules on sonogram were pathologically consistent with gathering areas of tumor cells and psammoma bodies, and the number of tumor cells decreased with increasing of distance from these areas. 2 lesions in the focal group had lymph vessel tumor emboli, mirroring the invasion of the surrounding parenchyma. The aggressiveness of DSVPTC lesions in the focal group was also reflected in nodular borders and the abundance of microcalcifications on sonogram. On the one hand, the borders of DSVPTC nodules were more poorly defined than cPTCs, corresponding to no capsule structural characteristics of DSVPTC, indicating the infiltrative growth of the tumor [24]. On the other hand, DSVPTC nodules had more microcalcifications than cPTCs, implying numerous psammoma bodies in pathology. Some scholars believed that the extensive psammoma bodies were calcified remnants of necrotic tumor cells in lymphatic vessels due to strong proliferative activity and invasiveness [24]. Nevertheless, the above two points are not enough to distinguish DSVPTC nodules from cPTC nodules and there are still limitations in identifying DSVPTC nodules by conventional ultrasound.

Given the above analysis, DSVPTC could present as the “snowstorm” pattern on sonogram, or hypoechoic solid nodules with vague borders and abundant microcalcifications. Both corresponded to aggressive lesions. Carcangiu ML et al. [25] thought the DSVPTC lesion developed from a localized nodule to the whole lobe scale, which may be linked to our two sonogram appearances. However, this view still needs further study to be confirmed.

The sonogram of DSVPTC can be confused with Hashimoto’s thyroiditis because both share the appearance of heterogeneous background. In our study, 80% of DSVPTC cases had a heterogenous background, while only 33% of cPTC cases had it. From one aspect, stromal fibrosis and lymphocyte infiltration are evident in DSVPTC, leading to a heterogeneous background like Hashimoto’s thyroiditis. On account of this, some scholars believed it was difficult to distinguish DSVPTC from Hashimoto’s thyroiditis on sonograms, resulting in delayed diagnosis of DSVPTC until the lobe was enlarged [26]. For another, DSVPTC is likely to be associated with Hashimoto’s thyroiditis. In a study by Joung JY et al. [27], 60% DSVPTC cases were complicated with Hashimoto’s thyroiditis. Spinelli C et al. [9] found 56% patients had coexistent Hashimoto’s thyroiditis. According to our results, the proportion of DSVPTC patients with pathologically Hashimoto’s thyroiditis was higher than that of cPTC (70.0%: 31.0%). Therefore, attention should be paid to distinguishing these two diseases by ultrasound, and great emphasis should be given to the heterogenous background in unilateral lobe to diagnose DSVPTC since both lobe involvement is common in Hashimoto’s disease.

Notably, in the DSVPTC lesions, 2 sonographically suspicious nodules were verified to be fibrotic nodules by pathology, which increased the risk of misdiagnosis. These 2 fibrotic nodules shared the similar ultrasound appearance, including hypoechogenicity and irregular margins as malignant nodules. One even had a taller-than-wide shape, and the other had microcalcifications. We have never seen such fibrotic nodules in DSVPTC reported before. We speculate that the fibrotic process began after the tumor invasion, as some psammoma bodies were left inside the fibrotic area. In clinical practice, fibrotic nodules in DSVPTC can pose a dilemma when choosing the site of fine needle aspiration. To increase the accuracy, we suggest selecting the parenchyma outside the nodules, especially that with a relatively high concentration of microcalcifications for puncture, in addition to the nodular area for suspicious DSVPTC patients.

All 10 DSVPTC cases in our study had cervical lymph node metastasis, with 80% shown on ultrasound. Previous studies also reported a high incidence of cervical lymph node metastasis of DSVPTC, with a rate of 68% [28] and an average number of 4.5 per case [7]. Kazaure HS et al. [29] found that cervical lymph node metastasis was more common among 261 DSVPTCs than among 42,904 cPTCs (72.2% vs. 56.3%). Some scholars believed that the widespread invasion of the thyroid lymphatic duct led to the propensity of lymph node metastasis [13]. In our study, the sonogram features of metastatic cervical lymph nodes of DSVPTC were similar to those of cPTC, including loss of central hilar echo, cystic change, calcification and cortical hyperechogenicity. Chen CC et al. [10] considered numerous microcalcifications in cervical lymph nodes as a crucial feature to discern DSVPTC. However, only 1 case in our study showed lymph nodes of that kind, which was not statistically significant in comparison with cPTC. One case in our study showed anechoic area in a metastatic cervical lymph node, contradicting the conclusion drawn by Zhu B et al. [30] that cystic change was scarcely seen in metastatic lymph nodes of DSVPTC. Overall, although the metastatic lymph nodes of DSVPTC were more common, the sonogram was not specific.

The study does have some limitations. First of all, it was a retrospective study. The results were inevitably affected by the operator’s preference, although the videos and images were interpreted by two radiologists to avoid intraobserver bias. Second, the sample size was small due to the low incidence of DSVPTC and the insufficient data. A multicenter study covering a larger population is needed. Third, we did not include other conventional variants of PTC, such as follicular variant, in the control group.

In conclusion, DSVPTC can present as the “snowstorm” pattern on sonogram indicating the psammoma bodies diffusely distributed in the lobe, or simply as hypoechoic solid nodules with vague borders and abundant microcalcifications suggesting an ongoing invasion. The lesions of both manifestations are aggressive and require the same attention from the surgeons. Nevertheless, it is difficult to diagnose DSVPTC via conventional ultrasound due to the following reasons: the sonogram of DSVPTC is easily confused with that of Hashimoto’s thyroiditis, the fibrotic nodules in DSVPTC lesions share a similar appearance with malignant thyroid nodules on sonogram, and the metastatic cervical lymph nodes do not have specific sonographic characteristics. Therefore, fine needle aspiration under the guide of ultrasound is necessary.

### Electronic supplementary material

Below is the link to the electronic supplementary material.

Supplementary Material 1. Additional file 1. Ultrasonographic images of other 7 DSVPTC cases

## Data Availability

Most of the data generated or analyzed during this study are included in the article [and its Additional file]. Other data, which were used under license for the current study, are not publicly available due to restrictions on the availability, but are available from the corresponding author (jianchu.li@163.com) on reasonable request.
