# Supplementary material for: Sonographic characteristics of diffuse sclerosing variant of papillary thyroid carcinoma with histopathological correlation: a preliminary study
Source: Orphanet J Rare Dis. 2024 Mar 26;19:136. doi: 10.1186/s13023-023-02867-3 (PMC10967076; doi:10.1186/s13023-023-02867-3)
Supplement: Supplementary file 1 — Supplementary Material 1. Additional file 1. Ultrasonographic images of other 7 DSVPTC cases [file 13023_2023_2867_MOESM1_ESM.pdf]

Figure S1 Ultrasonographic image of DSVPTC Case 1

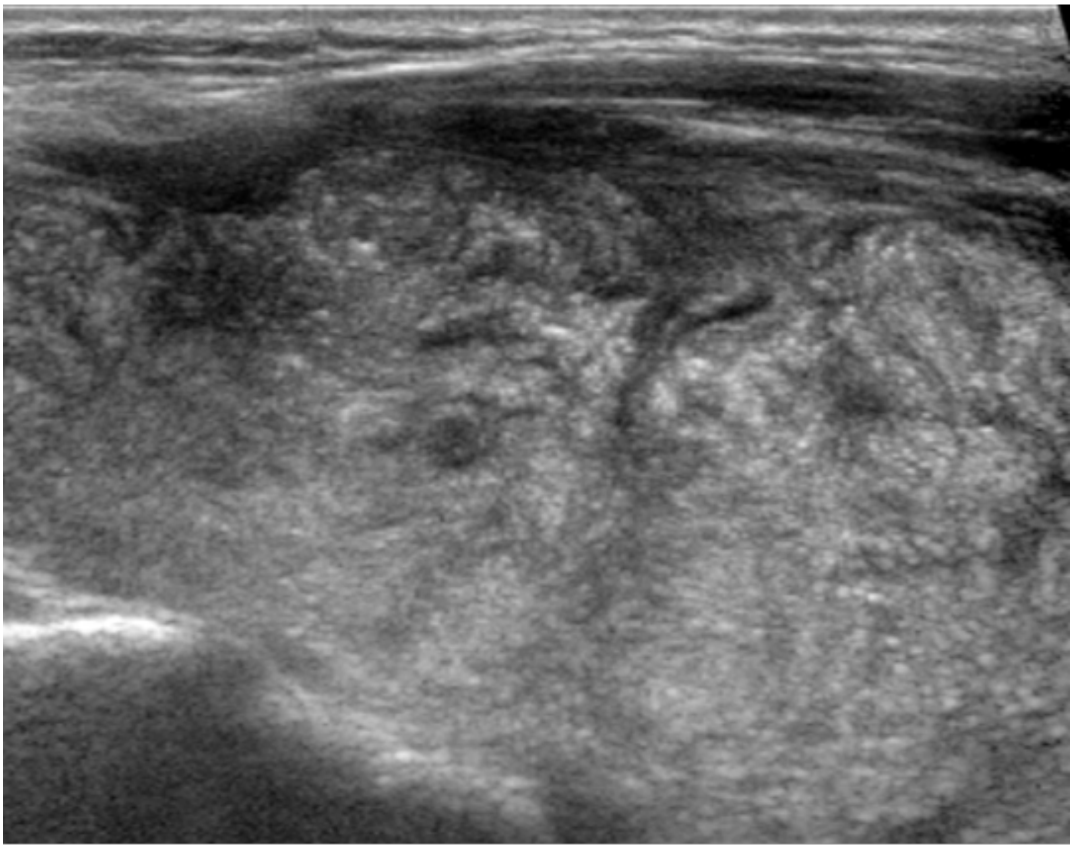

Numerous microcalcifications in the whole lobe like “snowstorm” without nodules in the longitudinal section of the thyroid.

Figure S2 Ultrasonographic image of DSVPTC Case 2

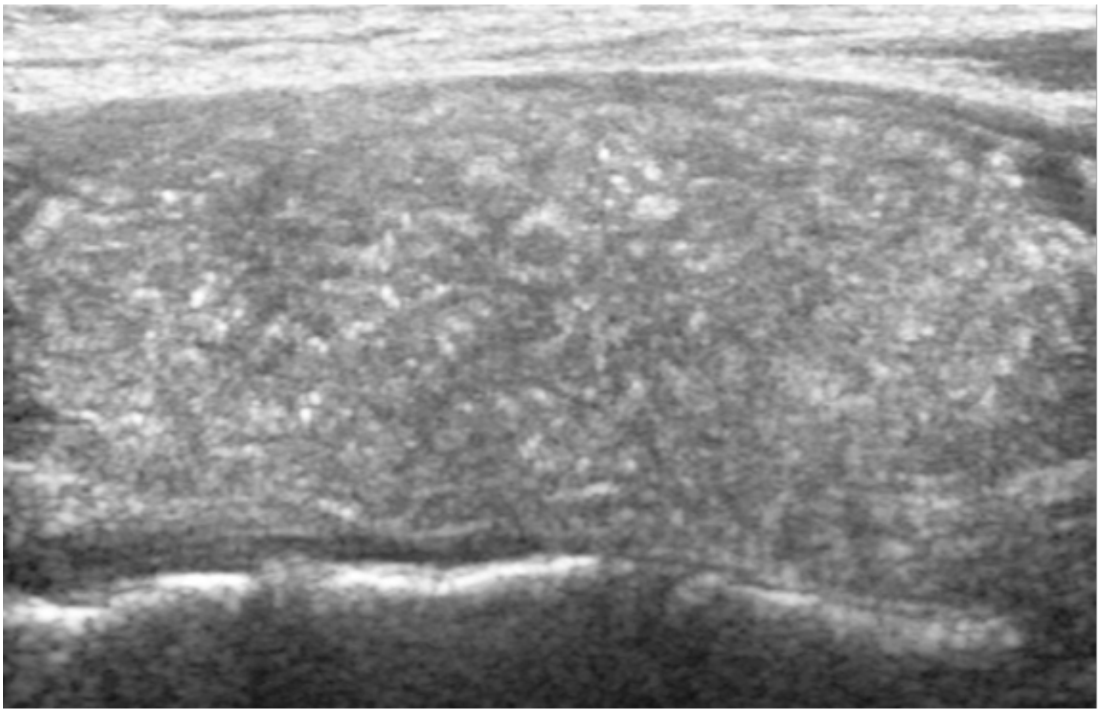

Numerous microcalcifications in the whole lobe like “snowstorm” without nodules in the longitudinal section of the thyroid.

Figure S3 Ultrasonographic image of DSVPTC Case 3

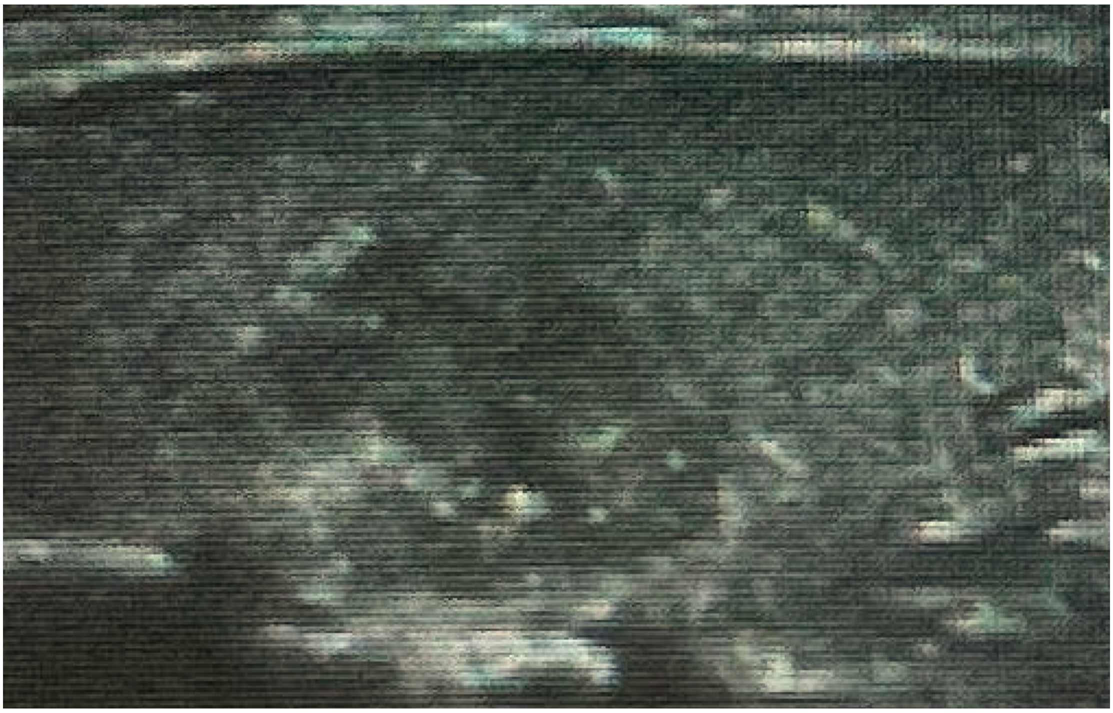

Numerous microcalcifications in the whole lobe like “snowstorm” without nodules in the longitudinal section of the thyroid (photograph of the thermal paper).

Figure S4 Ultrasonographic image of DSVPTC Case 5

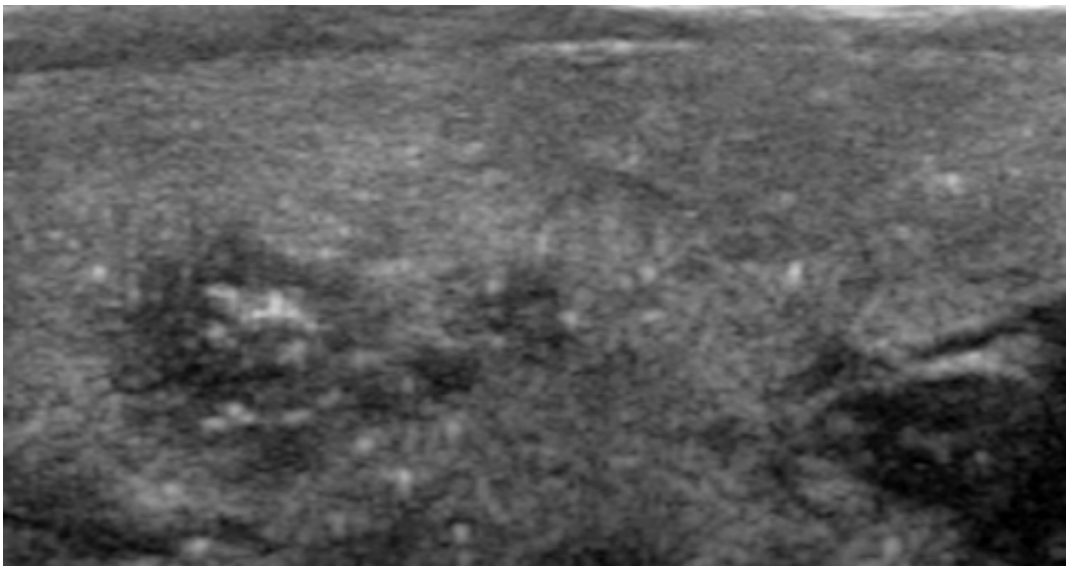

Numerous microcalcifications in the whole lobe with nodules of vague border and multiple microcalcifications in the longitudinal section of the thyroid.

Figure S5 Ultrasonographic image of DSVPTC Case 7

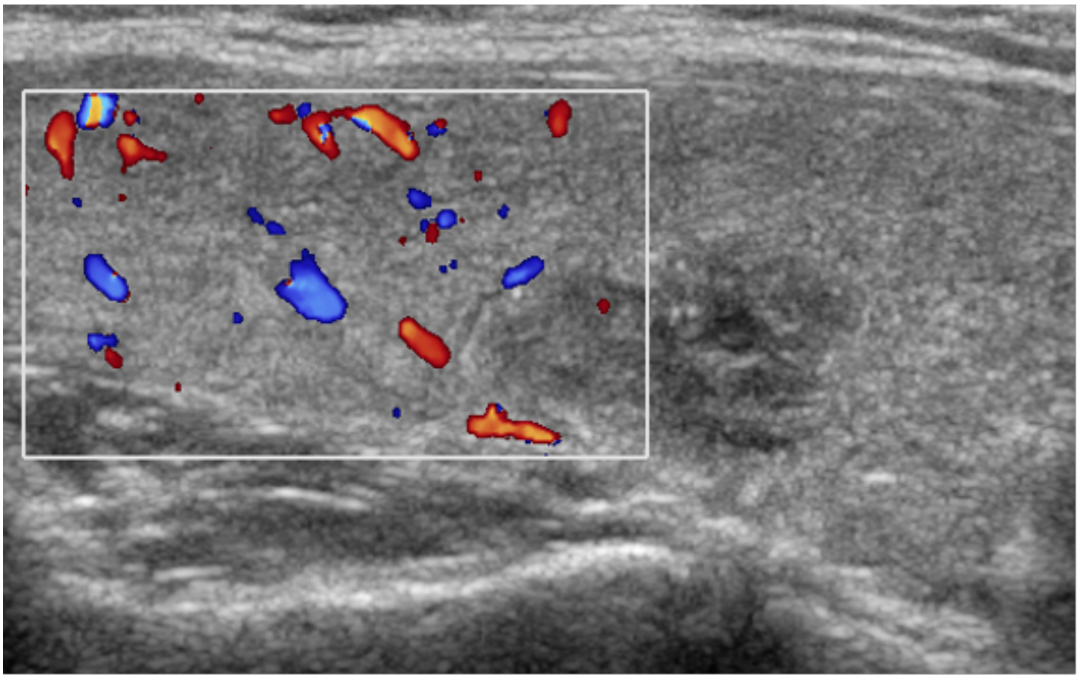

A hypoechoic nodule with vague border and multiple microcalcifications in the lobe with heterogenous background in longitudinal section of the thyroid.

Figure S6 Ultrasonographic image of DSVPTC Case 8

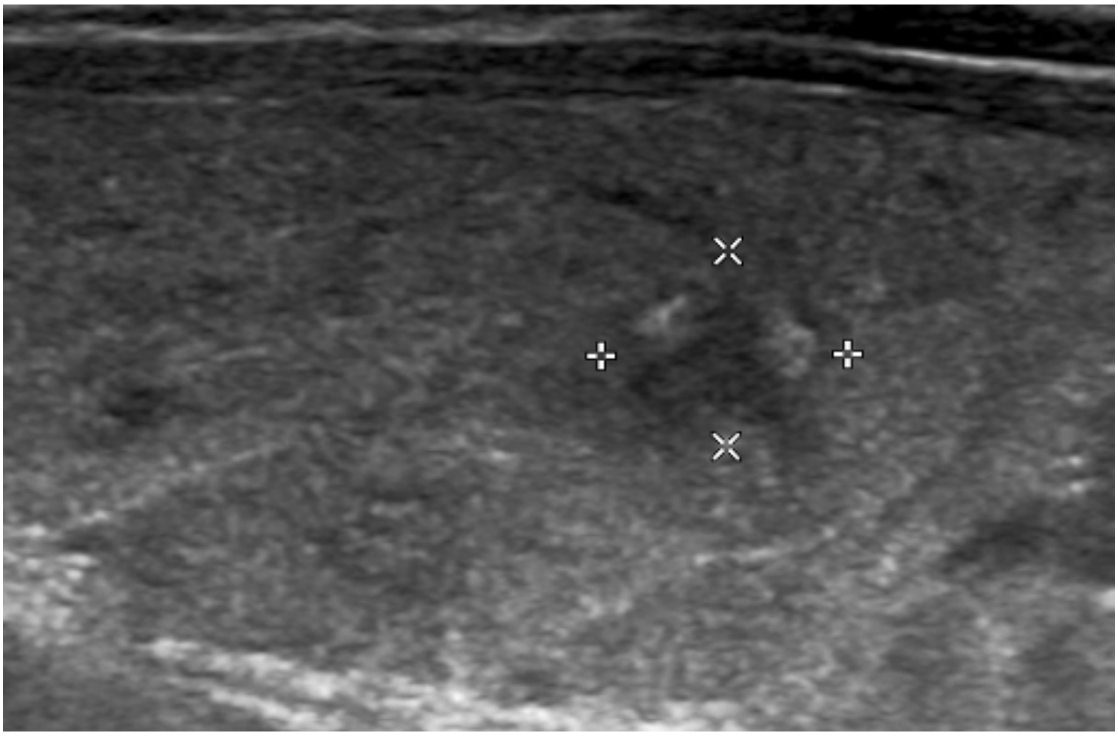

A hypoechoic nodule with vague border and several microcalcifications in the lobe with heterogenous background in longitudinal section of the thyroid.

Figure S7 Ultrasonographic image of DSVPTC Case 10

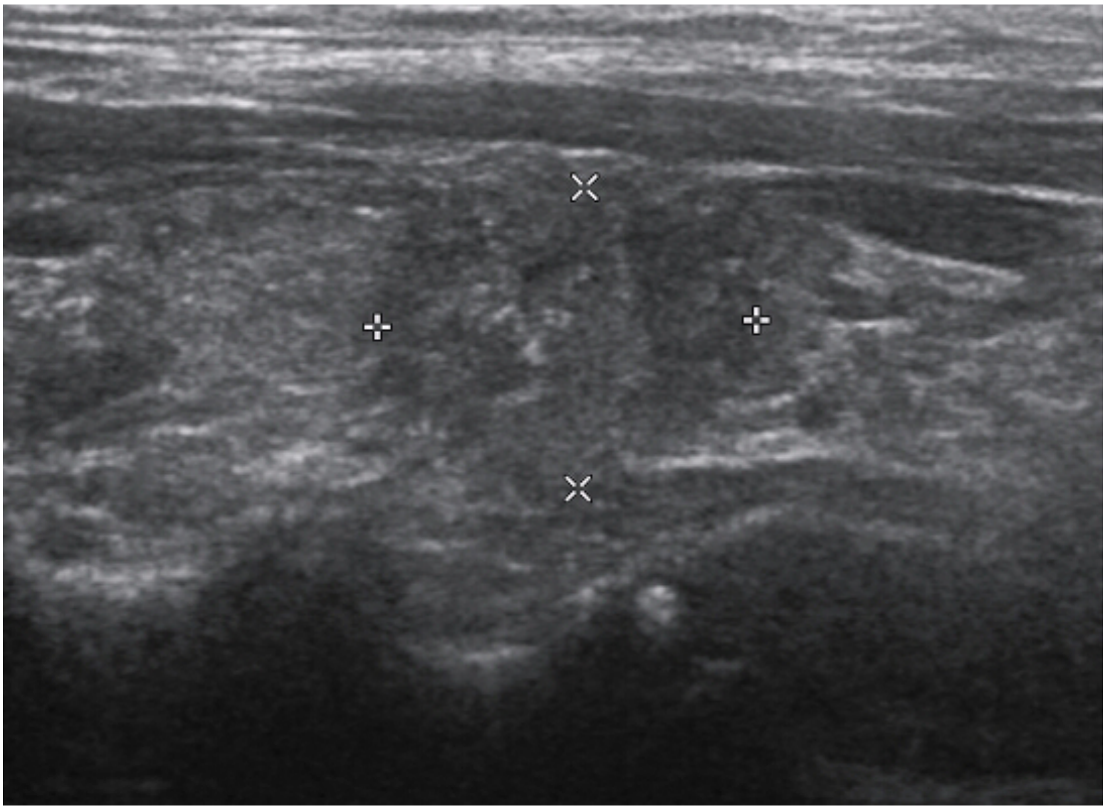

A hypoechoic nodule with vague border and several microcalcifications in the lobe with heterogenous background in longitudinal section of the thyroid.
